# Incidence rate and management of diaphragmatic injury during laparoscopic nephrectomies: single-center experience

**DOI:** 10.1093/jscr/rjac127

**Published:** 2022-06-09

**Authors:** Raffaele Baio, Giovanni Molisso, Christian Caruana, Oliviero Intilla, Umberto Di Mauro, Umberto Pane, Antonio Campitelli, Francesca Pentimalli, Roberto Sanseverino

**Affiliations:** Department of Medicine and Surgery “Scuola Medica Salernitana”, University of Salerno, Baronissi, Salerno, Italy; Department of Urology, Umberto I, Nocera Inferiore, Salerno, Italy; Department of Chemistry, University of Malta, Msida, Malta; Department of Urology, Umberto I, Nocera Inferiore, Salerno, Italy; Department of Urology, Umberto I, Nocera Inferiore, Salerno, Italy; Department of Urology, Umberto I, Nocera Inferiore, Salerno, Italy; Department of Urology, Umberto I, Nocera Inferiore, Salerno, Italy; Department of Clinical Pathology and Clinical Biochemistry, National Cancer Institute of Naples “Fondazione Giovanni Pascale”, Naples, Italy; Department of Urology, Umberto I, Nocera Inferiore, Salerno, Italy

## Abstract

Pneumothorax is a rare complication in laparoscopic renal surgery. However, due to the increasing renal pathologies managed by laparoscopic technique, this infrequent complication is a potential risk. We investigated the incidence rate of this complication in our experience of laparoscopic renal surgery, taking into account the laparoscopic approach, the type of intervention, the character of the pathology (neoplastic or other), the site of the intervention, as well as the localization of the lesion (in case of malignant pathology). About 384 laparoscopic nephrectomies were reviewed at our institution, with a total of four cases (1.04%) of diaphragmatic injury. Diaphragmatic repair was always carried out by intracorporeal suturing, with no complications. Repair of diaphragmatic injuries should always be attempted with intracorporeal suture since this is a safe and effective technique. Then, although in the retroperitoneal approach pneumothorax is more likely, our experience has shown that transperitoneal access is not free from this complication.

## INTRODUCTION

Inadvertent pleural injury may occur during open flank surgery for renal or adrenal disease. These injuries are usually suture repaired primarily with simultaneous evacuation of pleural air. Operative breach of the diaphragm is uncommon during laparoscopic renal and adrenal surgery [1–3]. However, with the widespread use of laparoscopy (for its clear advantages over open surgery) and the increasing surgical pathologies managed with this technique, there is a potential for an increased risk of carbon dioxide pneumothorax due to diaphragmatic injury. Capnothorax associated with laparoscopic surgery is different from air pneumothorax but, if such an injury is recognized intraoperatively, it should be repaired laparoscopically in a manner duplicating the principles of open surgery. We report the incidence and the laparoscopic repair for inadvertent diaphragm injury during upper abdominal urological laparoscopy.

## MATERIALS AND METHODS

**Figure 1:**
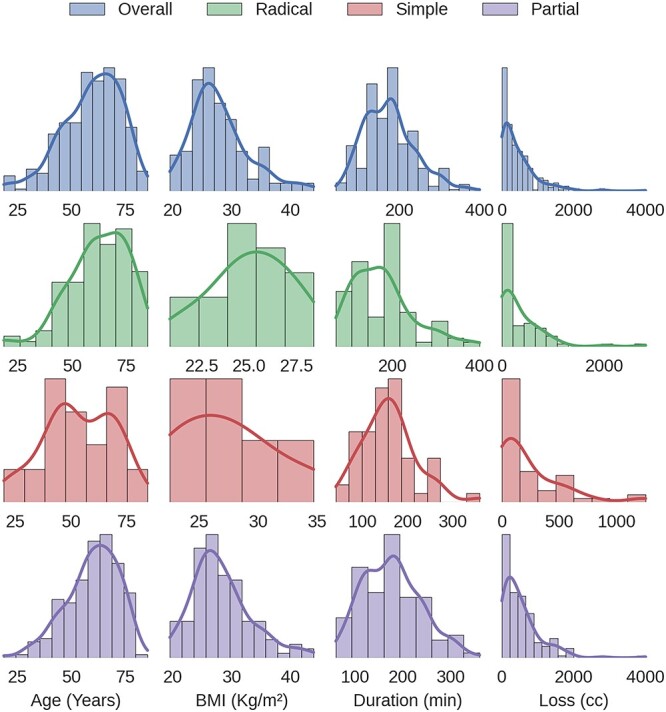
Distribution of the patient’s age, BMI, time of surgery and blood loss for all kidney surgeries and for each type of kidney surgery individually.

**Figure 2:**
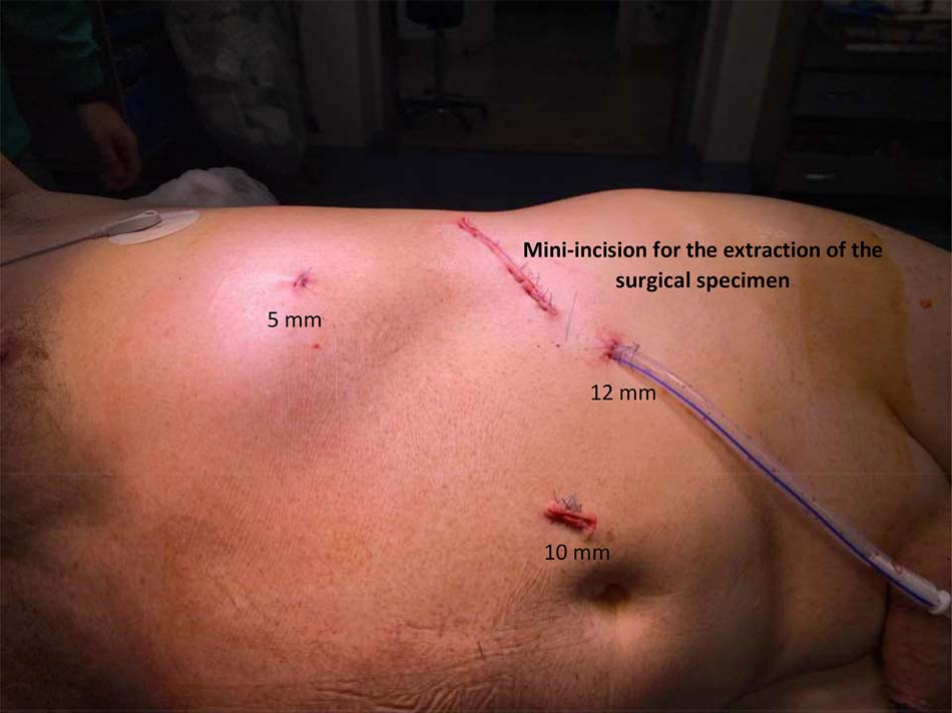
Usual working port configuration for nephrectomy. The left 5 mm port incision is always placed over the lower ribs in case of left radical or simple nephrectomy. However, in the case of nephroureterectomy, we prefer to use a 10 mm trocar in that position (rather than a 5 mm trocar) in an attempt to move the camera from a different point of view. This strategy allows us to better perform the dissection of the ureter near the bladder.

The records of 384 laparoscopic kidney operations were reviewed. All procedures were performed by the same surgeon (R.S.) and included: 65 simple nephrectomies, 115 radical nephrectomies and 204 partial nephrectomies. A total of four cases (1.04%) of diaphragmatic injury were found. Mean patient age was 59.7 years (range from 18.5 to 85.3). Operative and clinical records were reviewed and patients’ outcomes were evaluated. Figure 1 shows the distribution of the patient’s age, BMI, time of surgery and blood loss, considering all kidney surgeries and each type of kidney surgery individually. For diaphragmatic repair, no additional trocars were needed. In all cases, usual working port configuration for nephrectomy, as shown in Fig. 2, allowed intracorporeal suturing. However, if necessary, an additional 5 mm port was placed on left or right flank, respectively. In all cases of inadvertent injury, the pleural breach was identified intraoperatively (due to noticeable billowing of the diaphragm), followed by direct laparoscopic visualization of the injury. Laparoscopic suture repair of the diaphragm replicated the technique and repaired laparoscopically with interrupted 0-Vicryl sutures, whereas pneumoperitoneum was decreased in 12 mmHg and the anesthesiologist administered a large inspiratory breath. Criteria used for chest tube placement was pneumothorax greater than 20% of lung volume or associated with hemodynamic or ventilatory changes.

## RESULTS

A total of four cases (1.04%) of diaphragmatic injury were recorded during 384 laparoscopic nephrectomies. In all cases, the cause of pleural lesion was iatrogenic injury to the diaphragm. Two of these lesions occurred during partial nephrectomy, one during radical nephrectomy and another during simple nephrectomy. All four cases of diaphragmatic lesion occurred in the presence of a transperitoneal access and all in the course of left nephrectomy. As regards the pathology, in only one case the pathology was of a benign nature (being hydropyonephrosis). As regards the localization of the lesion, the distribution of the three malignant pathologies was as follows: one case with localization of the lower hemi-kidney and two cases with lesion of the upper pole of the kidney. Diaphragmatic repair was always carried out by intracorporeal suturing. All patients evolved uneventfully. Table 1 summarizes the injury specifications and operative management.

**Table 1:** Injury specifications and operative management

Case	Surgery	Side of Surgery	Type of Laparoscopic Approach	Type of Pathology	Localitation of Pathology	Repair Method
1	Partial nephrectomy	Left side	Transperitoneal approach	Cancer	Upper pole of kidney	Intracorporeal suture
2	Partial nephrectomy	Left side	Transperitoneal approach	Cancer	Upper pole of kidney	Intracorporeal suture
3	Radical nephrectomy	Left side	Transperitoneal approach	Cancer	Lower hemi-kidney	Intracorporeal suture
4	Simple nephrectomy	Left side	Transperitoneal approach	Hydropyonephrosis	—	Intracorporeal suture

## DISCUSSION

In laparoscopic renal and adrenal surgery, iatrogenic injury of the diaphragm is an uncommon event; in effect, it does not exceed 0.6% in the largest series [2]. As further confirmation of this, in 2001, Soulie *et al*. reported 19 urological complications in 350 laparoscopic procedures (5.4%) [4] but none of these patients showed diaphragmatic injury, highlighting its rarity. The occasional occurrence of this complication is due to the clear separation that exists between the kidneys and the diaphragm [5]. However, with the advances made in laparoscopic renal and adrenal surgery, more surgeons have expanded the limits for laparoscopy by attempting very demanding procedures. This may sustain or even increase the incidence of iatrogenic diaphragmatic injuries. It is noteworthy that our series reflects the experience of a single surgeon that has surpassed the learning curve of standardized techniques (OAC). Obesity, large tumors, inflammatory intestinal pathologies, previous surgeries and chemotherapy are some of the factors that can facilitate the occurrence of diaphragmatic lesions. However, adrenal surgery by itself has an inherent risk for diaphragmatic injury because the adrenal gland is juxtaposed against the diaphragm. Diaphragmatic injury can originate from improper trocar placement or direct contact with monopolar electrocautery or harmonic scalpel [2]. When the retroperitoneal approach is preferred for renal or adrenal surgery, improper trocar placement can easily lead to diaphragm injury [2]. The lesion can appear as an evident tear of the diaphragm or be invisible to the surgeon’s inspection and be alerted by changes in patient cardiopulmonary status (such as changes in auscultation, end inspiratory pressure, blood pressure and arterial blood gasses) [1]. In these cases, the anesthesiologist involvement is decisive in the diagnosis and timing of repair. In addition, an undetected injury may become evident by the floppy diaphragm sign, in which the diaphragm billows inferior with any degree of abdominal desufflation, reflecting the loss of negative pressure within the diaphragm [6]. All of our patients with inadvertent injury demonstrated undue billowing of the diaphragm, which prompted the rapid diagnosis of the diaphragmatic complication. So, this complication was recognized by the operating surgeon in all cases. None of our patients had hemodynamic instability as a result of the injury. To avoid diaphragmatic injury, care must be taken when large renal masses are dissected and during the mobilization of intra-abdominal structures for kidney exposure. Considering only single center experiences on this topic, our report is the second largest series after the work published by Castillo *et al*. [7]. Techniques of laparoscopic repair of the diaphragm have been described in the urological literature. In a multi-institutional review of 1765 patients, Del Pizzo *et al*. reported on eight patients who underwent laparoscopic repair of an iatrogenically injured diaphragm [2], using an EndoStitch device or freehand laparoscopic suturing with interrupted two-zero sutures. Before tying the last stitch, pleural gas was evacuated using a large inspiratory breath or a laparoscopic suction device. Upon the completion of repair, CO_2_ was aspirated from the pleural space using an intercostal 6 Fr catheter that was introduced by the Seldinger technique. In one patient (12%) a chest tube was necessary to evacuate residual pneumothorax. Fugita inserted a catheter into the diaphragmatic defect through the abdominal wall under laparoscopic vision [8]. No mention was made of whether the abdomen was desufflated before tying down the suture or whether the pleural space was evacuated under water seal. Similar to what was described by Del Pizzo [2] and by Castillo [7], we also chose interrupted sutures for the laparoscopic repair regardless of lesion size and location. Several reports confirm the feasibility of diaphragmatic repair by means of intracorporeal suturing [1, 9]. We believe that diaphragm suturing must always be attempted due to the simplicity and reliability of this technique. Although continuous suture tends to be faster, particularly for long defects and especially in laparoscopy, the risk of dehiscence is greater if the suture material breaks. For this reason, we prefer to perform an interrupted suture for its advantage of having a high tensile strength (although it takes a relatively long time to be placed). Nevertheless there has been one successful report of diaphragmatic injury repair without the use of stitches [10]. This was achieved by employing a matrix gel and a thrombin solution (Floseal) with interposition of the omentum over a 1 cm diaphragmatic lesion. The authors refer to their technique as a suitable option for small lesions. To reach an effective repair of the diaphragm, air must be evacuated before the stitches are secured by means of either a suction device or the administration of a long forced inspiratory breath. In addition, repair of diaphragmatic injury has to be timed according to patient parameters. When the patient is in stable condition surgery can continue and the injury may be addressed at the end of the procedure. In cases of large tumors that may obstruct the surgeon’s direct access to the lesion, surgical specimen should be removed first to ease repair. Nevertheless we think that if possible, the diaphragm injury should be repaired without delay. This was the case in all of our patients in which early recognition of diaphragmatic injury allowed for a prompt repair without the interference of the surgery. Pneumothorax greater than 20% of lung volume or associated with hemodynamic or ventilatory changes is managed with thoracostomy [9]. Pleural lesions produced by trocar placement or important residual capnothorax may also warrant thoracostomy. CO_2_ used for pneumoperitoneum is readily eliminated through the lungs and, compared with air, it has higher solubility and increased diffusion coefficient; this allows a greater amount of molecules to diffuse across a membrane in a given time. This explains why capnothorax usually resolves spontaneously and allows for expectant management in patients without hemodynamic instability [9]. Although the precise time frame for such resolution is not clear, Venkatesh *et al*. reported spontaneous resolution of a symptomatic pneumothorax within 3 h [9]. As such, whether to intervene depends on the individual clinical scenario and surgical judgment. Abreu *et al*. reported a higher incidence of gas collections associated with the retroperitoneal over the transperitoneal approach (6.6% vs. 0.7%) [3]. However, they concluded that asymptomatic, subclinical, spontaneously resolving gas collections in the chest are more common with retroperitoneoscopy but the incidence of symptomatic or serious thoracic complications is similar between transperitoneal and retroperitoneal laparoscopy [11]. We did not observe injuries from direct trocar entry in our series; this can be explained by the fact that we prefer the transperitoneal to the retroperitoneal approach for renal or adrenal surgery.

## CONCLUSIONS

Primary laparoscopic repair/reconstruction of iatrogenic injury of the diaphragm should always be attempted with intracorporeal suture since this is a safe and effective technique. We believe that most laparoscopic surgeons with reasonable experience can reproduce this technique and iatrogenic injury to the diaphragm should not necessitate open conversion. Then, although when the retroperitoneal approach is preferred for renal or adrenal surgery, improper trocar placement can easily lead to diaphragm injury, and our experience has shown that transperitoneal access is not free from this complication.
